# The mystery of COVID-19 reinfections: A global systematic review and meta-analysis

**DOI:** 10.1016/j.amsu.2021.103130

**Published:** 2021-12-04

**Authors:** Rubaid Azhar Dhillon, Mohammad Aadil Qamar, Jaleed Ahmed Gilani, Omar Irfan, Usama Waqar, Mir Ibrahim Sajid, Syed Faisal Mahmood

**Affiliations:** a Medical College, Riphah International University, Rawalpindi, Punjab, Pakistan; b Ziauddin Medical College, Karachi, Sindh, Pakistan; c Aga Khan University, Karachi, Sindh, Pakistan; d Amaris Consulting, Toronto, Ontario, Canada; e Medical College, Aga Khan University, Karachi, Sindh, Pakistan; f Section of Infectious Diseases, Aga Khan University, Karachi, Sindh, Pakistan

**Keywords:** COVID-19, Reinfection, LMIC, HIC, Reactivation

## Abstract

**Background:**

As the COVID-19 pandemic rages on, reports on disparities in vaccine roll out alongside COVID-19 reinfection have been emerging. We conducted a systematic review to assess the determinants and disease spectrum of COVID-19 reinfection.

**Materials and methods:**

A comprehensive search covering relevant databases was conducted for observational studies reporting Polymerase Chain Reaction (PCR) confirmed infection and reinfection cases. A quality assessment tool developed by the National Institute of Health (NIH) for the assessment of case series was utilized. Meta-analyses were performed using RevMan 5.3 for pooled proportions of findings in first infection and reinfection with a 95% confidence interval (CI).

**Results:**

Eighty-one studies reporting 577 cases were included from 22 countries. The mean age of patients was 46.2 ± 18.9 years and 179 (31.0%) cases of comorbidities were reported. The average time duration between first infection and reinfection was 63.6 ± 48.9 days. During first infection and reinfection, fever was the most common symptom (41.4% and 36.4%, respectively) whilst anti-viral therapy was the most common treatment regimen administered (44.5% and 43.0%, respectively). Comparable odds of symptomatic presentation and management were reported for the two infections. However, a higher Intensive Care Unit (ICU) admission rate was observed in reinfection compared to first infection (10 vs 3). Ten deaths were reported with respiratory failure being the most common cause of death (7/10 deaths).

**Conclusion:**

Our findings support immunization practices given increased ICU admissions and mortality in reinfections. Our cohort serves as a guide for clinicians and authorities in devising an optimal strategy for controlling the pandemic. (249 words)

## Highlights

• COVID-19 reinfection is not a phenomenon to be taken lightly.
• 81 studies from 22 countries reported PCR confirmed infection and re-infection.
• The average reported time duration between first infection and reinfection was 63.6 (±48.9) days.
• A higher ICU admission rate was observed in reinfection compared to first infection.
• Complete recovery rate after reinfection stood at 97.9%.

## 1 Introduction

The coronavirus, through its rapid spread and emerging variants, started in Wuhan in December 2019 [[1], [2], [3]]. It was declared a global pandemic in March 2020 and continues to persist as a public healthcare emergency [4].Since its onset, the virus has infected more than 266 million individuals globally and has resulted in more than 5.2 million deaths till date [5].

Currently, there are seven types of coronaviruses known to infect humans: 4 are seasonal and cause limited upper respiratory tract infections, whereas the other 3, namely SARS coronavirus (SARS-CoV-1), Middle East respiratory syndrome (MERS), and SARS-CoV-2 have been reported to cause severe disease [6]. The cause of the current pandemic, SARS COV-2, binds to the angiotensin-converting enzyme 2 receptors using a receptor-binding domain in its spike protein for cell entry and ultimately, resulting in a respiratory syndrome [[6], [7], [8]]. The currently available vaccines target the spike protein [6]. However, mutations in the spike protein have been implicated in the reduction of small, but significant, efficacy of vaccines [9,10] highlighting the scale of the challenge COVID-19 places to the world.

Even though the world is heralding the development of new vaccines as a potential way forward for a healthier, more “COVID-free” time, the concern of reinfection, recurrence, and mutant variants still continues to loom. The Center for Disease Control and Prevention (CDC), in their October 27, 2020 report [11], raised concerns over reactivation of the disease and requested the public to maintain infection control measures such as, wearing a mask in public, maintaining a six feet distance, regular hand washing, and avoiding crowded spaces.

Since Tillett et al. [12] reported the first confirmed case of COVID-19 reinfection from the USA, several other authors have also described their patient experiences of viral recurrence. The reason for this recurrence and its potential public health implications is a question that warrants explanation. Iwasaki et al. [13] reason that perhaps a scant antibody response following the first infection could be the cause of relapse. They emphasize ascertaining a degree of specificity of the antibody (anti-nucleocapsid vs anti-spike antibody) at the time of reinfection, as well as determining the immune correlations of protection.

The public concern of whether vaccines could be a potential cure for the viral outbreak remains to be explained, with the obvious apprehension of whether a separate vaccine would need to be developed for every variant of the virus. To date, there have been four variants with significant mutations in the spike protein that have gained widespread surveillance: B.1.1.7 (VOC 202012/01 or 20B/501Y.V1) which originated in the UK, B.1.351 (20H/501Y.V2) which originated from the Republic of South Africa, and P.1 (B.1.1.28.1) which was reported in travelers coming from Brazil [14]. A fourth double mutant variant of concern, labeled B.1.617 originating from India, was reported in March 2021, considered to be a cause of the massive rise in infections in India during its second wave [15]. Currently, it is the most dominant strain worldwide according to the World Health Organization (WHO) [16]. A fifth mutant variant of concern, B.1.1.529 (Omicron), has just recently been announced by the WHO [17] with a preprint retrospective analysis of routine epidemiological data from South Africa showing this variant to be associated with an increased risk of reinfection [18].

The objective of this review, in addition to providing comprehensive evidence on COVID-19 reinfections including both pediatric and adult cases, is a unique comparison of the first infection and the reinfection disease spectrum, management, and outcomes.

## 2 Methods

The protocol of the review is registered with The International Prospective Register of Systematic Reviews (PROSPERO) CRD42021239816 [19]. The review is reported in accordance with the Preferred Reporting Items for Systematic Reviews and Meta-Analyses (PRISMA) 2020 guidelines [20]. The AMSTAR-2 checklist [21] was also used to assess this study which determined this study to be a high-quality review.

### 2.1 Search methods

An exhaustive literature review was conducted on major databases: PubMed, WHO COVID-19 Database, Embase, China National Knowledge Infrastructure (CNKI) Database, Google Scholar, manual searches of leading medical journals, and a pre-print server, medRxiv, covering the timeline of January 1st, 2020, to March 16th, 2021. The following keywords were used to conduct the search: COVID-19 and derivatives, reinfection, relapse and reactivation, as shown in S1 Table. Complementary searches were conducted in the Johns Hopkins Health Resource, Chinese, and US CDC Library. No language restrictions were applied. Key reference lists were additionally screened for more studies.

### 2.2 Selection strategy

Observational studies (cohorts, case series, and case reports) reporting laboratory‐confirmed COVID-19 (RT-PCR) first infection and reinfection were considered for inclusion. Review articles, commentaries, and letters not presenting any original data were excluded. Covidence software (2016 edition) was utilized for screening by two reviewers, independently and in duplicate. Any discrepancies in selection were resolved by a third independent author.

### 2.3 Data extraction and analysis

The shortlisted articles were then extracted, independently and in duplicate, on a structured data form by two reviewers. The information extracted from each study was as follows: Author names, date of publication, country, setting of the study, type of study, number of patients and age group, patient information (age, gender and comorbidities), clinical evaluation (presenting symptoms in both infections, travel/exposure history and infected family members), diagnostic tests (nasopharyngeal swabs, antibody tests and timelines for initial infection and reinfection), radiographic findings, therapeutic regimen (medications, isolation, and plasma therapy), outcomes for both infections (hospitalization, Intensive Care Unit (ICU) admission, complications, discharge, and death) and antibody status after both infections. Disaggregated data by age groups (children and adults) was extracted, where available.

Categorical data were summarized as counts and proportions. The pooled proportions of reported findings were calculated using Review Manager 5.3's random-effects model. I^2^ was calculated to examine statistical heterogeneity. The clinical features and outcomes were compared accordingly between first infection and reinfection using pooled proportions and their 95% CI's, which was supplemented by an odds ratio (OR).

### 2.4 Quality assessment

Individual study quality was evaluated independently by the review authors using the quality assessment tools developed by the National Heart Lung and Brain Institute (NHLBI) [22]. The study quality was scored out of 8 by two independent authors, based on clarity of study objectives, case definition, consecutive inclusion of cases, comparability of included patients, definition and measurement of outcomes, length of follow-up, statistical methods and results. Studies with score 6–8 were graded to be of good quality, 4–5 considered fair quality and <4 scores was considered to be of poor quality.

## 3 Results

**Fig. 1:**
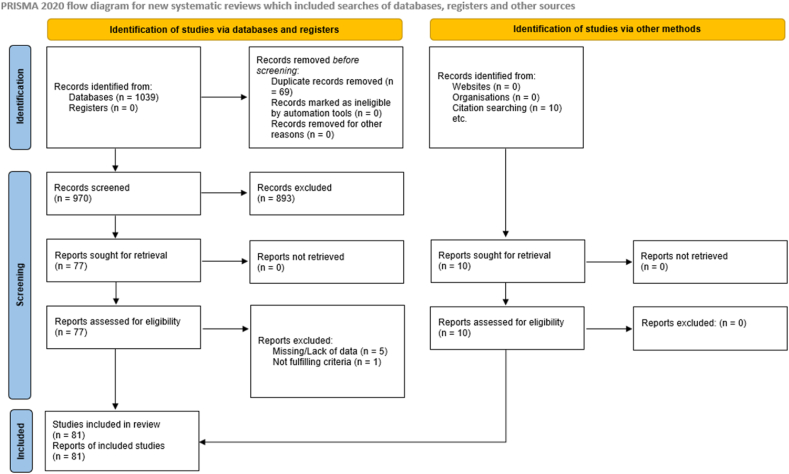
PRISMA Flow diagram of study selection process.

The comprehensive literature search yielded a total of 1039 studies from the databases. Before screening, 69 of these studies were removed due to duplication. The remainder 970 studies from the databases were then screened and a further 893 records were excluded. The remaining 77 records were sought for retrieval and then assessed for eligibility. Six studies were excluded as they either presented with overlapping or missing data (n = 5) and/or did not fulfill the inclusion criteria (n = 1). Likewise, 10 studies were identified via citation searching. These 10 were sought for retrieval and then assessed for eligibility [12, [23], [24], [25], [26], [27], [28], [29], [30], [31], [32], [33], [34], [35], [36], [37], [38], [39], [40], [41], [42], [43], [44], [45], [46], [47], [48], [49], [50], [51], [52], [53], [54], [55], [56], [57], [58], [59], [60], [61], [62], [63], [64], [65], [66], [67], [68], [69], [70], [71], [72], [73], [74], [75], [76], [77], [78], [79], [80], [81], [82], [83], [84], [85], [86], [87], [88], [89], [90], [91], [92], [93], [94], [95], [96], [97], [98], [99], [100], [101], [102]]. None of these were excluded and overall, 81 studies were found to be eligible according to our inclusion criteria. The characteristics of these 81 included studies is shown in S2 Table. An overview of the detailed systematic study selection process is presented in the PRISMA flow diagram (Fig. 1).

Through consultation with a professional librarian, a search was conducted in leading medical journals and through duplication and screening processes, studies were selected.

In terms of study types, 45 studies (55.6%) were case reports, and 36 studies (44.4%) were case series. Studies were reported from 22 countries. Forty-eight out of 81 studies were reported from low-and-middle-income countries (LMICs). More than one-third of the studies were reported from China (28/81, 34.6%); almost one-sixth were from the USA (11/81, 13.6%), accounting together for about half of the studies (39/81, 48.1%) included in our review. The global distribution of studies is shown in Table 1 and S1 Fig .

**Table 1:** Distribution of studies (N = 81) and reinfection cases (N = 577) according to country of origin included in the review.

Country	Number of studies	Number of reinfection cases
Belgium	2	2
Brazil	7	45
China	28	423
Colombia	1	1
Ecuador	1	1
France	5	19
Hong Kong	3	3
India	4	8
Iraq	1	26
Israel	1	1
Italy	4	4
Iran	1	9
Netherlands	1	1
Pakistan	1	1
Peru	1	1
Portugal	1	1
Qatar	1	1
UK	3	8
USA	11	14
Turkey	2	3
Switzerland	1	1
Korea	1	4
Total	81	577

### 3.1 Demographics and epidemiology

A total of 577 cases with a mean age of 46.2 ± 18.9 years (range 3.0–91.0 years) were included in the study. The gender of study cases was noted to be 45.8% males and 53.7% females. Of the 577 cases, approximately one-third of the cases (n = 179, 31.0%) were reported to have at least one comorbidity.

Reports of having a positive contact history with close contact or a family member with COVID-19 were found in 87 (15.1%) cases (S2 Table and Table 2). Across 76 studies, the average reported time duration between first infection and reinfection was 63.6 ± 48.9 days (range 11.0–210.0 days).

**Table 2:** Demographics of patients.

Variable	Mean Proportion (%)
**Age (Years)**	46.2 ± 18.9 years (range 3.0–91.0 years)
**Gender**	
Male	264 (45.8)
Female	310 (53.7)
Not specified	3 (0.5)
**Contact History Positive**	87 (15.1)
**Underlying Comorbidities**	179 (31.0)
**Average time duration between first infection and reinfection**	63.6 ± 48.9 days (range 11.0–210.0 days)

### 3.2 Clinical features during first infection and reinfection

Around three-fourth of our cases were categorized as being mildly symptomatic with only 10 cases being classified as severe to critical by their respective studies for disease severity in the first infection. The total number of asymptomatic cases during the first infection was 53 (9.2%) with an increase to 184 (31.9%) cases noted during reinfection (S2 Table). Tian M et al. [83], reported the highest number of asymptomatic cases (20/577, 3.5%) during the first infection whereas An J et al. [28], with 27 cases (4.7%) reported the highest number of asymptomatic cases during reinfection. The presence of antibodies was also reported for the total 577 cases, wherein 323 (56.0%) and 364 (63.0%) cases were detected to be positive during first infection and reinfection, respectively.

**Fig. 2:**
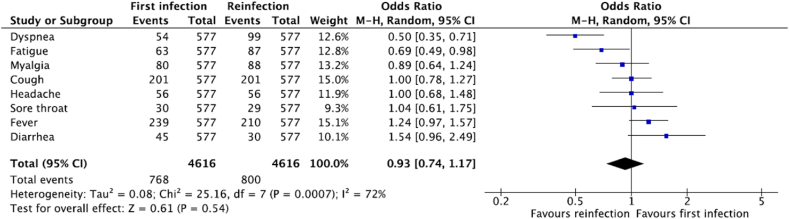
Clinical features of First Infection and Reinfection (N = 577).

The most common presenting symptoms among patients in the first infection were fever (n = 239, 41.4%) and cough (n = 201, 34.8%), which then accounted for 36.4% (n = 210) and 34.8% (n = 201) of cases, respectively, during reinfection. Myalgia was reported in 80 (13.9%) cases during first infection which increased to 88 (15.3%) cases during reinfection. The frequencies and odds ratio of other reported signs and symptoms are listed in Fig. 2.

Regarding the radiological imaging during first infection of the 577 cases, almost one-quarter (146/577, 25.3%) had not reported, or did not have, any kind of chest imaging done. Out of those who reported, only 25 (4.3%) cases had a normal finding, whereas 295 (51.1%) cases reported an abnormality with Chen et al. [38], reporting the highest occurrence of radiological abnormalities (70 out of 81 cases, 86.4%).

### 3.3 Management of first infection and reinfection

**Fig. 3:**
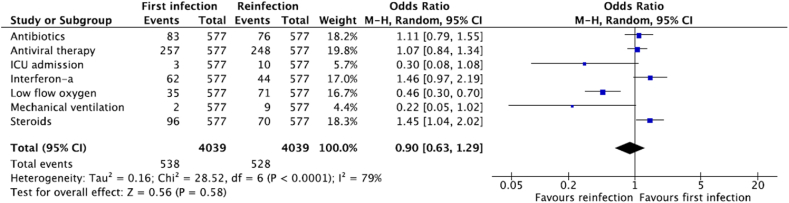
Management of first infection and reinfection (N = 577).

The most administered treatment, as reported by the study results, was antiviral therapy accounting for 44.5% (n = 257) and 43.0% (n = 248) of cases during first infection and reinfection, respectively. Administration of antibiotics was lower at 14.4% (n = 83) and 13.2% (n = 76) for first infection and reinfection respectively (Fig. 3).

In the study by He et al. [53], 60% of patients were administered steroids during both infections, which was reported to be the highest use of steroids among studies reported to date. However, in our data, the overall use of steroids was 16.6% (n = 96) and 12.1% (n = 70) during the respective infections. Traditional Chinese medicine and interferon administration were reported at 13.9% (n = 80) and 10.7% (n = 62) during the first infection and 26.7% (n = 154) and 7.6% (n = 44) during reinfections, respectively.

Lastly, our results show that the use of low flow oxygen was reported in 6.1% (n = 35) of cases during the first infection, which then doubled to 12.3% (n = 71) of cases during reinfection (Fig. 3). Out of the total studies, reports of ICU admission and mechanical ventilation were relatively low being 0.5% (n = 3) and 0.3% (n = 2) during first infection, respectively, compared to 1.7% (n = 10) and 1.6% (n = 9) during reinfection, respectively. This was contradictory to a higher number of asymptomatic cases observed in the reinfection phase, implicating for a possible need of an Individual Patient Data (IPD) analysis in future studies.

Comparable odds of symptomatic presentation (OR:0.93, 95%CI: 0.74–1.17) and management (OR:0.90, 95%CI: 0.63–1.29) were observed in the first infection compared to reinfection when meta-analyzed, as shown in Figs. 2 and 3. Although a higher event of management was observed in the first infection, due to the individual weight of the studies, the overall OR favored first infection and was less than 1.

### 3.4 Outcomes of first infection and reinfection

Complete recovery rate after reinfection stood at 97.9% (565 cases) with a total of 10 (1.8%) deaths. The outcome status was unknown for 2 cases (0.3%) (S2 Table). The eight expired cases were elderly (72–91 years old; 1 male and 7 females) and 2 cases were middle-aged adults (44 and 54 years old; both males). Seven cases had comorbidities involving multiple organ systems whilst three suffered from hypertension and the remaining one had an underlying malignancy. Respiratory failure was the most common cause of death (seven out of ten deaths).

### 3.5 Pediatric (0–19 years) reinfection cases

Out of 577 cases, disaggregated data for 24 pediatric (0–18 years) cases was available. Disaggregation reported positive contact histories in 7 cases (29.2%) with only 1 (4.2%) reporting a comorbidity. A total of 7 cases (29.2%) were asymptomatic followed by fever (n = 4, 16.7%) and cough (n = 3, 12.5%) in first infection whereas during reinfection, asymptomatic presentation (n = 7, 29.2%) was followed by cough (n = 4, 16.7%) and then fever (n = 3, 12.5%). With 9 abnormal chest X-ray findings (37.5%), the most frequently used management modalities during first infection and reinfection were anti-viral (n = 12) and traditional Chinese Medicine (n = 12), respectively. All patient outcomes were reported as recovered.

### 3.6 Quality assessment of included studies

Seventy-two studies were determined to be of good quality while nine studies were of fair quality (S3 Table). Studies were primarily downgraded for unclear study objectives [74], incomplete case definition [27,39,59,77,78,81,88,94], non-consecutive subject recruitment [30,38,41,45,49,50,[55], [56], [57],^60^,^82^,^90^,^91^,^97^,^101^], incomparable subjects [90], inadequate length of follow-up [54,74], inadequate description of statistical methods [50,57,91] and inadequate description of results [50,89]. The most common cause for downgrading studies was non-consecutive recruitment, which raised concerns that the included sample could be biased towards a more severe presentation or included more individuals undergoing routine screening.

## 4 Discussion

Both the developing and developed worlds are still battling the spread of COVID-19. A major concern that needs to be addressed is the appearance of reinfections in previously recovered COVID-19 patients.

Our review is the first, and largest, systematic review covering COVID-19 reinfection cases from over 22 countries, raising questions concerning vaccination and exploring a specific set of determinants that can facilitate reinfection in recovered individuals. Similar systematic reviews on COVID-19 reinfection have been conducted previously [103,104] but none of those studies, or any conducted so far in the literature, have been as extensive as this review in terms of analyzing the clinical information between first infection and reinfection whilst covering a wide range of international and regional databases.

One of the major strengths of this review is the substantial timeframe that it covers: January 2020 to March 2021, spanning a total of 81 studies with a widespread distribution of High-Income Countries (HICs) and LMICs to differentiate features of reinfection cases as per different settings. In addition, adult cases were separated from pediatric cases to differentiate between clinical features and identify the optimal treatment management strategies as per varying age groups. Furthermore, case reports and case series included in our study were quality assessed with 72 out of 81 studies reported to be of good quality. An analysis of only pediatric reinfection cases was also conducted in this review. Good prognosis and lower morbidity were reported in pediatric cases, similar to the general COVID-19 disease course in the pediatric population [105]. Therefore, we suggest public health campaigns targeting people of younger age as they are at similar risk of reinfection as adults, to ensure elimination of complacency and enforcement of protective measures, such as face masks and social distancing.

A broad distribution was seen among severity of the first infection compared to reinfection as well as management and number of symptomatic cases. The most-reported clinical symptoms in our review were fever (41.4%) and cough (34.8%) in first infection, with a frequency of 36.4% cases with fever and 34.8% cases with cough in reinfection, respectively. These findings are similar to a trend observed in the review on reinfections by Gidari et al. [106] In addition, the number of asymptomatic cases in our review increased from 9.2% in first infection to 31.9% in second infection, similar to findings reported in the review by Gidari et al. [106].

On the contrary, a higher requirement of ICU admission and mechanical ventilation was observed during reinfection in our review. A meta-analysis analysis of 123 cases by Vancsa et al. [107] showed that the second episode of SARS-COV-2 infection is more severe than the first if it happens within 60 days of the first positive PCR. This deems the necessity of IPD analysis as many of the larger case series report more severe cases which might skew the overall findings. A total of 10 deaths were reported in this review, all among reinfection cases. In all 10 cases, several comorbidities were present and all patients who were classified as the most severe were older, a similar trend seen in a study by Wang et al. [108]. Most of these patients died due to respiratory complications; similar effects of these comorbidities can be seen in other respiratory illnesses such as MERS-CoV [109]. The results from this review suggest that comorbidities and age play a major role in the outcome of critical patients.

The time duration between the first infection and reinfection has been a source of debate. Alinaghi et al. in their systematic review [104] estimated that antibodies from natural infection lasted 40 days, after which the chances of reinfection increased. The average time duration between first infection and reinfection in our review was 63.6 days. Wang et al. in their review noted it to be 76 days [110] whereas Manish et al. in their review [111] observed a lengthier duration of median 113.5 days. This variation in time duration outlines the need for vigilance when it comes to COVID-19 reinfection, especially considering waning antibodies.

Another observation made in our review was differences in the presence of antibodies during the first infection and reinfection. A recently published systematic review looked at antibody response following SARS-COV-2 infection across multiple studies [112]. They noted that 80% of patients developed IgM antibodies with antibodies being detected after a mean period of 7 days and declining after 27 days. Total 95% of patients developed IgG antibodies, with antibodies being detectable after a mean period of 12 days and started declining after 60 days. Likewise, IgA levels and neutralizing antibodies started declining after 30 days. In our review, 56.0% and 63.0% of the patients detected positive for antibodies during first infection and reinfection, respectively. Some evidence suggests that waning antibodies places individuals at a risk for reinfection, which may explain our finding of the time duration of first infection and reinfection being a mean of 63 days. The presence of antibodies could provide a protective role, but it does not specifically prevent reinfection as supported by findings in a systematic review by Piri et al. [113].

Whilst our review did not analyze the cause of reinfection being due to different variants, they cannot be excluded. A review by Wang et al. [110] concluded that previous COVID-19 reinfection did not confer total immunity and a second infection by a different variant was possible, with the second infection being more severe than the first. Even though most of the studies in our review predate the announcements of the new variants, however, given the ability of the virus to mutate at a rapid pace, some reinfection cases reported in our study could be due to the variants which would have resulted in more severity of reinfection. Whether waning antibodies or new variants were the source of reinfection is a question that should be explored further in future studies.

A recent systematic review by Azam et al. looked at the incidence of SARS-COV-2 positivity in patients who had recovered from COVID-19 [114]. They noted that younger patients and those with a longer initial infection were more likely to have recurrent positivity. A similar systematic review by Manish et al. on the assessment of SARS-COV-2 mutations in reinfections and persistent infections [111] noted it to be challenging to differentiate between reinfection and persistent recurrent infection, concluding that the former happened in immunocompetent individuals and the latter happened in immunocompromised individuals. Furthermore, this phenomenon was associated with a faster viral evolution and mutation resulting in the creation of new variants. Lastly, another systematic review by Hoang on the risk factors associated with re-positive viral RNA after recovery from COVID-19 [115] postulated that the re-positive viral RNA seen in their review likely added to the evidence that viral relapse was a cause of COVID-19 recurrence.

At this time, public health initiatives aimed at removing complacency are the need of the hour, and one of the key messages that need to be given is that reinfection is a reality, and vaccines along with social distancing remain the key in fighting the pandemic. A recently published online longitudinal survey [116] in 23 countries of high, middle and low income, across 4 continents with over 1 million participants provides hope in this regard, as it identified that the intention to vaccinate amongst the general public is at an all-time high, with the major issue not being vaccine hesitancy but instead, a shortage of vaccines. A recent report of a 4-month surveillance of mass immunization in Israel [117] notes two doses of the Pfizer BioNTech mRNA COVID-19 vaccine to be highly effective (95.3%; 95% CI 94.9–95.7) against SARS-CoV-2 infection and mitigated COVID-19-related hospitalizations, severe disease, and death, including those caused by variants such as the B.1.1.7 SARS-CoV-2.

Whilst the world is still in the process of getting vaccinated, data needs to be collected on patients, in the long run, to analyze whether vaccination has any correlation with reinfection cases and to further investigate the average time needed by the various vaccines to achieve their desired efficacies. We hope that governments across the world seize this moment and take steps to ensure equitable distribution of vaccines so that the world can finally step out of the long shadow cast by the COVID-19 pandemic.

However, a few questions remain. Firstly, would immunity conferred by the first infection protect individuals from a serious disease process in the reinfection phase? And secondly, does reinfection imply that individuals who are already vaccinated experience a more severe COVID-19 infection? Further studies should be done to answer this very important key facet of reinfection arising in COVID-19 especially in the context of breakthrough infections being reported around the world in vaccinated individuals, with the B.1.617 variant implicated to be the predominant cause [118,119].

### 4.1 Limitations

This review has some limitations, such as the small sample sizes analyzed from each country except for China that had 73% (n = 423) of the total included cases. The majority of these cases were reported from Wuhan or the Hubei province, where the gross domestic product per capita is less than half of that of Beijing and Shanghai [120]. Therefore, the findings of studies from China may be generalizable to the socioeconomic and health development status of other middle-income countries and not to high-income nations. This review can be improved by sampling larger series and including IPD, if available, to predict the outcome of COVID-19 illness based off epidemiological trends dramatically reducing hospitalization time, given the lack of sufficient healthcare resources in low-middle income countries. Therefore, a selection bias remains when considering LMICs where admitted hospital patients could be in a more critical state reporting a higher mortality rate. Also, treatment approaches to COVID-19 have altered dramatically over the time of these studies and may have changed many of the outcomes in the first and second infections reported in this study. This may have accounted for some of the differences in ICU treatment or clinical outcome. Likewise, the number of unique outcomes, be it ICU admissions or deaths, were minimal with only about 10 cases or less total in either the primary infection or recurrence (of 577 total). That makes examining differences between first and second infections difficult and clearly predisposes to a type-two error, especially given the variations in case definitions between the studies.

## 5 Conclusion

COVID-19 first infections and reinfections observe a similar clinical spectrum and management regimen with a slightly higher severity reported during reinfection in the form of requirement for mechanical ventilation and ICU admission. There lies a need for much closer scrutiny of reinfections globally with individual patient data analysis to derive determinants of reinfection incidence and disposition to a severe infection.

## Ethical approval

This study did not need any ethical approval being a systematic review and meta-analysis of publicly available data.

## Sources of funding for your research

This study had no sources of funding.

## Author contribution

OI conceptualized the study design and objectives. OI, MAQ and RAD drafted the study protocol, conducted the literature search, study screening, selection and data extraction. OI, MAQ and RAD designed the data collection instrument, collected data and carried out data analysis. OI, MAQ, RAD, JAG, MIS and UW drafted the initial manuscript. JAG, OI, MAQ critically reviewed and revised the final manuscript. SFM is the guarantor and critically reviewed the manuscript. All authors approve the final manuscript as submitted for publication.

## Consent

Not applicable.

## Registration of research studies

1. Name of the registry: International Prospective Register of Systematic Reviews (PROSPERO).

2. Unique Identifying number or registration ID: CRD42021239816.

3. Hyperlink to your specific registration (must be publicly accessible and will be checked): https://www.crd.york.ac.uk/prospero/display_record.php?ID=CRD42021239816.

## Guarantor

Dr Syed Faisal Mahmood is the guarantor.

## Provenance and peer review

Not commissioned, externally peer-reviewed.

## Data availability statement

Data is available upon request to the corresponding author.

## Declaration of competing interest

The authors have no conflicts of interest to declare.

## Figures and Tables

**S1 Fig:**
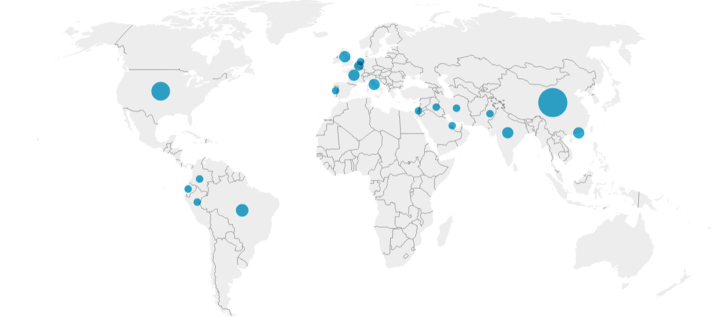
Global map of distribution of the studies included (N = 81) in the review.

## Appendix A Supplementary data

S1 Table Search Strategy for MEDLINE. S1 Table

S2 Table eCharacteristics of included studies (N = 81). S2 Table

S3 Table Quality assessment of case reports and case series (N = 81). S3 Table
