# Supplementary material for: The mystery of COVID-19 reinfections: A global systematic review and meta-analysis
Source: Ann Med Surg (Lond). 2021 Dec 4;72:103130. doi: 10.1016/j.amsu.2021.103130 (PMC8642249; doi:10.1016/j.amsu.2021.103130)
Supplement: Multimedia component 5 [file mmc5.pdf]

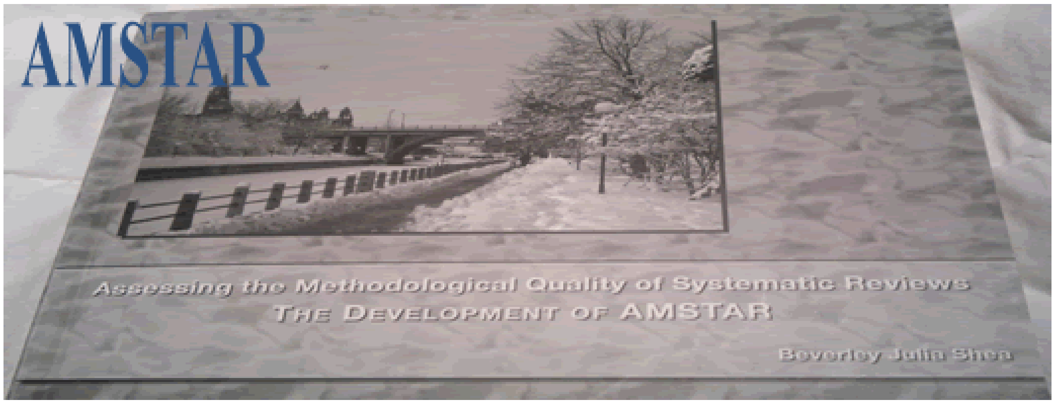

AMSTAR 2 Results

Printer Friendly Version

Article Name: The Mystery of COVID-19 Reinfections

You are currently logged on as Guest. You need to be logged on as a member to submit your score.
Log On

The Mystery of COVID-19 Reinfections is a High quality review

1. Did the research questions and inclusion criteria for the review include the components of PICO?

Yes
Yes
Yes
Yes
Yes

---

2. Did the report of the review contain an explicit statement that the review methods were established prior to the conduct of the review and did the report justify any significant deviations from the protocol?

YesYesYesYesYesYesYesYes

---

3. Did the review authors explain their selection of the study designs for inclusion in the review?

Yes

Yes

---

4. Did the review authors use a comprehensive literature search strategy?

Yes
Yes
Yes
Yes
Yes
Yes
Yes
Yes
Yes

---

5. Did the review authors perform study selection in duplicate?

Yes
Yes

---

6. Did the review authors perform data extraction in duplicate?

Yes
Yes

**7. Did the review authors provide a list of excluded studies and justify the exclusions?**	Yes<br>Yes<br>Yes
**8. Did the review authors describe the included studies in adequate detail?**	Yes<br>Yes<br>Yes<br>Yes<br>Yes<br>Yes<br>Yes<br>Yes<br>Yes<br>Yes<br>Yes
**9. Did the review authors use a satisfactory technique for assessing the risk of bias (RoB) in individual studies that were included in the review?**<br>**RCT**	0
**NRSI**	Partial Yes<br>Yes<br>Yes<br>Yes
**10. Did the review authors report on the sources of funding for the studies included in the review?**	Yes<br>Yes
**11. If meta-analysis was performed did the review authors use appropriate methods for statistical combination of results?**<br>**RCT**	0
**NRSI**	Yes<br>Yes<br>Yes
**12. If meta-analysis was performed, did the review authors assess the potential impact of RoB in individual studies on the results of the meta-analysis or other evidence synthesis?**	No
**13. Did the review authors account for RoB in individual studies when interpreting/ discussing the results of the review?**	Yes<br>Yes
**14. Did the review authors provide a satisfactory explanation for, and discussion of, any heterogeneity observed in the results of the review?**	Yes<br>Yes

**15. If they performed quantitative synthesis did the review authors carry out an adequate investigation of publication bias (small study bias) and discuss its likely impact on the results of the review?**	Yes<br>Yes
**16. Did the review authors report any potential sources of conflict of interest, including any funding they received for conducting the review?**	Yes<br>Yes

To cite this tool: Shea BJ, Reeves BC, Wells G, Thuku M, Hamel C, Moran J, Moher D, Tugwell P, Welch V, Kristjansson E, Henry DA. AMSTAR 2: a critical appraisal tool for systematic reviews that include randomised or non-randomised studies of healthcare interventions, or both. BMJ. 2017 Sep 21;358:j4008.

<< Back

Copyright © 2021 AMSTAR All Rights Reserved |
